# Integrating bioinformatics and experimental validation to unveil disulfidptosis-related lncRNAs as prognostic biomarker and therapeutic target in hepatocellular carcinoma

**DOI:** 10.1186/s12935-023-03208-x

**Published:** 2024-01-13

**Authors:** Lixia Xu, Shu Chen, Qiaoqiao Li, Xinyi Chen, Yuan Xu, Yongjian Zhou, Juan Li, Zhixian Guo, Jiyuan Xing, Di Chen

**Affiliations:** 1 Department of Infectious Diseases, The First Affiliated Hospital of Zhengzhou University, Zhengzhou, Henan 450052 China; 2 School of Basic Medical Sciences, Xinxiang Medical University, Xinxiang, Henan 453003 China; 3 The Second Affiliated Hospital of Chongqing Medical University, No. 76 Linjiang Road, Chongqing, 400010 China; 4 The First Clinical Medical College, Gannan Medical University, Ganzhou, Jiangxi 341000 China; 5 Department of Neurosurgery, The First Affiliated Hospital of Zhengzhou University, Zhengzhou, Henan 450052 China

**Keywords:** Disulfidptosis, Long non-coding RNA, Hepatocellular carcinoma, Prognostic signature, Immune microenvironment, TMCC1-AS1

## Abstract

**Background:**

Hepatocellular carcinoma (HCC) stands as a prevalent malignancy globally, characterized by significant morbidity and mortality. Despite continuous advancements in the treatment of HCC, the prognosis of patients with this cancer remains unsatisfactory. This study aims at constructing a disulfidoptosis‑related long noncoding RNA (lncRNA) signature to probe the prognosis and personalized treatment of patients with HCC.

**Methods:**

The data of patients with HCC were extracted from The Cancer Genome Atlas (TCGA) databases. Univariate, multivariate, and least absolute selection operator Cox regression analyses were performed to build a disulfidptosis-related lncRNAs (DRLs) signature. Kaplan–Meier plots were used to evaluate the prognosis of the patients with HCC. Functional enrichment analysis was used to identify key DRLs-associated signaling pathways. Spearman’s rank correlation was used to elucidate the association between the DRLs signature and immune microenvironment. The function of TMCC1-AS1 in HCC was validated in two HCC cell lines (HEP3B and HEPG2).

**Results:**

We identified 11 prognostic DRLs from the TCGA dataset, three of which were selected to construct the prognostic signature of DRLs. We found that the survival time of low-risk patients was considerably longer than that of high-risk patients. We further observed that the composition and the function of immune cell subpopulations were significantly different between high- and low-risk groups. Additionally, we identified that sorafenib, 5-Fluorouracil, and doxorubicin displayed better responses in the low-score group than those in the high-score group, based on IC50 values. Finally, we confirmed that inhibition of TMCC1-AS1 impeded the proliferation, migration, and invasion of hepatocellular carcinoma cells.

**Conclusions:**

The DRL signatures have been shown to be a reliable prognostic and treatment response indicator in HCC patients. TMCC1-AS1 showed potential as a novel prognostic biomarker and therapeutic target for HCC.

**Supplementary Information:**

The online version contains supplementary material available at 10.1186/s12935-023-03208-x.

## Background

Primary liver cancer stands as a pervasive and lethal malignancy worldwide, posing grave threats to human life and health [1, 2]. Hepatocellular carcinoma (HCC) accounts for approximately 75–85% of primary liver cancers [3]. Currently, early surgical resection is still considered the first-line treatment to decrease the rate of mortality in patients with HCC [4, 5]. With continuous advancements at the medical level, new therapeutic options, such as interventional therapy, targeted therapy, and immunotherapy, have been proposed [5, 6]. However, the prognoses for HCC patients remain unfavorable, with a persistently poor 5-year survival rate [4]. The main factors leading to the poor prognosis are the insidious onset and the high heterogeneity of tumors, making it difficult to find a therapeutic target for HCC. Additionally, the infiltrative and disseminated nature of HCC tumors makes it practically impossible to completely remove the tumor by surgery, and the rapid drug resistance along with drug side effects also limit the treatment efficacy of drugs [2, 7]. Therefore, an in-depth exploration and understanding of the biological processes involved in the occurrence and progression of HCC is essential for the improvement of clinical diagnosis and treatment in patients with HCC.

Recent investigations have shed light on a distinctive form of programmed cell death known as disulfidptosis, which is triggered by the accumulation of reactive oxygen species and relentless lipid peroxidation induced by disulfide-dependent mechanisms [8, 9]. This disulfidptosis process leads to disulfide stress and ultimately culminates in cell death. Moreover, accumulating evidence shows that disulfidptosis is associated with the progression and prognosis of cancer [10]. For instance, Liu et al. demonstrated that susceptibility of the actin cytoskeleton to disulfide stress leads to disulfidoptosis, proposing a therapeutic avenue targeting disulfidoptosis for cancer treatment [8, 10]. Chen et al. constructed a disulfidptosis-related lncRNAs signature for predicting the prognosis and immunotherapy of glioma [11]. However, novel biomarkers linked to disulfidoptosis for HCC prognosis and therapy remain elusive. Thus, our dedication lies in pinpointing new biomarkers to advance targeted therapies for HCC patients through this innovative mode of cell death.

Long non-coding RNAs (lncRNAs) are non-coding RNAs with more than 200 nucleotides [12]. Recent studies suggest that lncRNAs are related to multiple biological processes in HCC, including cell proliferation, angiogenesis, and invasion, and thus are emerging as new targets for the diagnosis, treatment, and prognosis of HCC [13–15]. Additionally, the construction of lncRNA signatures has proven valuable in predicting the prognosis of HCC patients, offering novel clinical insights for guiding targeted treatment approaches [14]. For example, Xu et al. demonstrated that a ferroptosis-related nine-lncRNA signature can effectively predict prognosis and immune response in HCC [15]. However, the involvement of lncRNAs in the disulfidoptosis process of HCC remains obscure. The potential of disulfidoptosis-related lncRNA (DRLs) signatures as prognostic biomarkers for HCC patients has yet to be systematically evaluated.

In this study, we established a novel DRLs signature designed to predict the overall survival (OS) of HCC patients. Subsequently, we delved into the immune microenvironment of HCC, examined the participation of tumorigenesis pathways, and identified potential drugs for HCC treatment based on the prognostic signature. Furthermore, our findings underscored the functional relevance of TMCC1-AS1 in HCC progression, revealing that its inhibition resulted in suppressed cell proliferation, migration, and invasion. Collectively, this study enhances our comprehension of HCC prognosis and lays the groundwork for developing individualized therapeutic strategies.

## Methods

### Data acquisition and determination of prognostic DRLs

The RNA sequencing transcriptome data and clinical information of patients with HCC were retrieved from The Cancer Genome Atlas (TCGA) dataset (https://portal.gdc.cancer.gov/). To obviate statistical bias in our study, individuals lacking complete clinical information were excluded. Ultimately, 374 patients with HCC and 50 healthy individuals were included in subsequent analyses (last accessed: 6 May 2023). Ten disulfidptosis-related genes (GYS1, LRPPRC, NCKAP1, NDUFA11, NDUFS1, NUBPL, OXSM, RPN1, SLC3A2, and SLC7A11) were collected based on previously published studies [8–11, 16]. We performed Pearson correlation analysis with a threshold of Pearson’s *R* > 0.4 and *p* < 0.001 to assess the relationship between disulfidptosis-related genes and lncRNAs. Subsequently, univariate Cox regression analysis was performed to evaluate the prognostic significance of the DRLs (*p* < 0.001).

### Construction and validation of the DRL prognostic signature

The entire TCGA set was randomly divided into training and testing sets. The training set was used to establish the DRL signature, and the testing set along with the entire TCGA set was employed to validate the reliability of the signature. Subsequently, the R package “glmnet” was enlisted to establish the Least Absolute Shrinkage and Selection Operator (LASSO) Cox regression, incorporating a penalty parameter determined through 10-fold cross-validation and a significance threshold of 0.05. The computation formula for the risk score is expressed as follows: Risk score = Σ [Exp (lncRNA) × coef (lncRNA)]. Herein, Exp (lncRNA) signifies the expression levels of the included lncRNAs, while coef (lncRNA) denotes their respective regression coefficients. Based on the risk scores (with the median risk score used as a cutoff), all the HCC samples were separated into the low- and high-risk groups. The prognosis of patients with HCC was assessed by K-M curves and ROC curves.

### Independent prognostic analysis and establishment of a nomogram

Univariate and multivariate (*p* < 0.05) Cox regression analyses were conducted to confirm whether the prognostic signature can be used as a clinical prognostic predictor independent of other clinicopathological characteristics (age, gender, grade, and stage) in the patients with HCC using the R package “survival.” Additionally, a nomogram was established to predict the survival of patients with HCC via the R package “survival” and “regplot.” The accuracy of nomogram was estimated using the consistency index (C-index) and calibration curves.

### PCA and functional enrichment analysis

Principal component analysis (PCA) was performed using the R package “scatterplot3d” to weaken the dimensionality, identify the model, and visualize the high-dimensional data of the entire gene expression profiles, disulfidptosis-related genes (DRGs), DRLs, and risk model. The differentially expressed genes (DEGs) between the high- and low-risk groups were identified (|log2fold-change (FC)| > 1 and adjusted *p* < 0.05). Gene Ontology (GO) functional analyses, including cellular component (CC), molecular function (MF), biological processes (BP), and Kyoto Encyclopedia of Genes and Genomes (KEGG) pathway enrichment analyses, were performed on DEGs using the R package “clusterProfiler,” “org.Hs.e.g.db,” and “enrichplot.”

### Immune-related functional analysis and tumor mutation burden (TMB) analysis

The immune infiltration statuses were analyzed via the tools XCELL, TIMER, QUANTISEQ, MCPCOUNTER, EPIC, CIBERSORT-ABS, and CIBERSORT according to the profile of infiltration estimation for all TCGA tumors [17]. The differences in immune-related functions, infiltrating immune cells, and immune checkpoints between the low and high-risk groups were analyzed using the R package “ggpubr,” “reshape2,” and “ggplot2.” Additionally, we utilized the “maftools” package to examine and integrate the TCGA data and analyzed the difference in TMB between high- and low-risk groups.

### TIDE analysis and drug efficacy evaluation for HCC treatment

We utilized the tumor immunity dysfunction and exclusion (TIDE) algorithm to assess the differences in immunotherapy response between the low-risk and high-risk groups (http://tide.dfci.harvard.edu/) [18]. Furthermore, the half-maximal inhibitory concentration (IC50) was used to predict the sensitivity of patients with HCC to chemotherapeutic and targeted therapeutic agents. Screening of therapeutic drugs and observation of drug sensitivity using the R packages included “pRRophetic,” “limma,” “ggpubr,” and “ggplot2” with pFilter = 0.0001.

### Tumor samples collection

A total of eight HCC tissue specimens and eight corresponding normal liver samples were obtained from individuals undergoing surgical resection during the period spanning November 2022 to April 2023 at the First Affiliated Hospital of Zhengzhou University, situated in Henan, China. Following the surgical excision of tissue, the samples were promptly subjected to freezing in liquid nitrogen. The study garnered approval from the Ethics Committee of the First Affiliated Hospital of Zhengzhou University, aligning with the principles set forth in the Declaration of Helsinki.

### Cell culture and reverse transcription quantitative PCR (RT-qPCR)

The hepatocellular carcinoma cell lines (HEP3B and HEPG2) and normal liver control cell (NC) were procured from the National Collection of Authenticated Cell Cultures (Shang Hai, China). HEP3B and HEPG2 cells underwent cultivation in RPMI-1640 medium supplemented with 2 mM l-glutamine and 10% Fetal Bovine Serum (FBS) within a humidified incubator set at 37 °C with 5% CO2. Total cellular RNA was extracted using TRIzol reagent (Invitrogen, Carlsbad, CA, United States). Data normalization was achieved through glyceraldehyde-3-phosphate dehydrogenase (GAPDH) mRNA expression, and calculations were executed using the 2^(-ΔΔCT) method. The primer sequences for RT-qPCR analysis are provided in Supplementary Table S1.

### Cell transfection

Two siRNAs targeting TMCC1-AS1 (si-TMCC1-AS1) and a negative control (si-NC) were synthesized by GenePharma (Shanghai, China). Transfection of HEP3B and HEPG2 cells was carried out using si-TMCC1-AS1#1, si-TMCC1-AS1#2, and si-NC with lipofectamine® 3000 (Invitrogen, USA). After 24 h, the transfection efficiency was evaluated using RT-qPCR. The sequences of the siRNAs can be found in Supplementary Table S2.

### Cell counting kit-8 (CCK-8) assay

The HCC cells were seeded into 96-well plates at a density of 3 × 10^3^ cells per well. Subsequently, 10 μL of CCK-8 solution (Dojindo, Tokyo, Japan) was added to each well at 0, 24, 48, and 72 h, followed by a 2-hour incubation period. The absorbance of the cells at 450 nm was then measured using a SpectraMax i3x instrument (Molecular Devices, USA). After 72 h, the proliferation curve of the cells was constructed based on the absorbance values.

### Transwell migration and invasion assays

The migratory and invasive capacities of HCC cells were assessed using 24-well Transwell chambers with an 8 μm pore size (Corning, NY, USA). For the migration assay, 3 × 10^4^ HCC cells were placed in the top compartment containing 250 μL of serum-free medium, while the bottom compartment received 500 μL of medium with 10% FBS. After 48 h of culture, cotton swabs were employed to eliminate cells in the upper compartment. The cells traversing the filter were fixed with 95% ethanol, stained with a 0.5% crystal violet solution, and subsequently imaged and counted using a microscope (Olympus, Tokyo, Japan). In the invasion assay, prior to cell inoculation, the filter was coated with a layer of Matrigel (BD Biosciences, San Jose, CA, USA). The remaining procedures were analogous to those of the migration assay.

### Wound healing assay

Wound healing assays were executed following previously delineated protocols [19]. Briefly, cells were seeded in 6-well plates and incubated at 37 °C. With the cells were completely attached, we scraped the middle of the plate to form a wound and replaced the medium with serum-free medium. After 48 h, the coverage of the line was measured.

### Statistical analysis

The R software (version 4.1.3) was used for all statistical analyses and graph visualization. The classification variables in the training and testing sets were contrasted using the chi-square test. Student’s t-test or one-way ANOVA test was utilized to determine the differences between the high- and low-risk groups. The links between clinicopathological factors, risk score, immune check inhibitors, and immune infiltration levels were assessed using the Pearson correlation test. *P* < 0.05 was considered statistically significant.

## Results

### Identification of DRLs in HCC patients

**Fig. 1:**
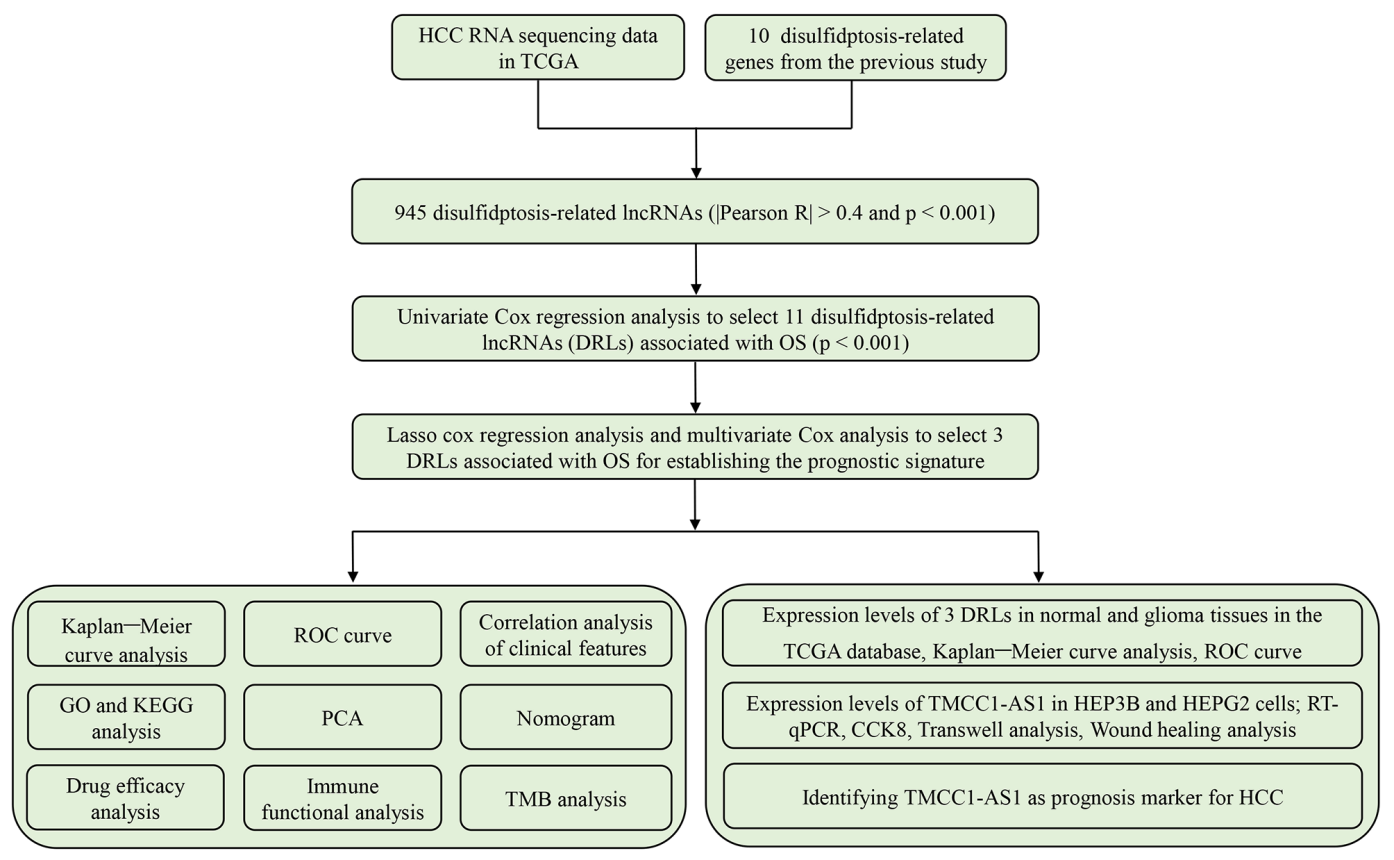
The flow diagram of the research process

A comprehensive flow diagram is depicted in Fig. 1. Initially, we gathered a total of 16,876 lncRNAs from the TCGA database’s HCC project and acquired 10 DRGs from previously published studies. Next, 945 DRLs were found by performing Pearson correlation analysis (|Pearson R| > 0.4 and *p* < 0.001) between lncRNAs and DRGs. Following the criteria of |log2 fold change (FC)| > 1 and *p* < 0.05, we obtained 750 differentially expressed DRLs. A heatmap was established to visualize the differential expression of DRLs between normal and tumor samples (Fig. S1A).

### Construction and validation of the DRLs prognostic signature

**Fig. 2:**
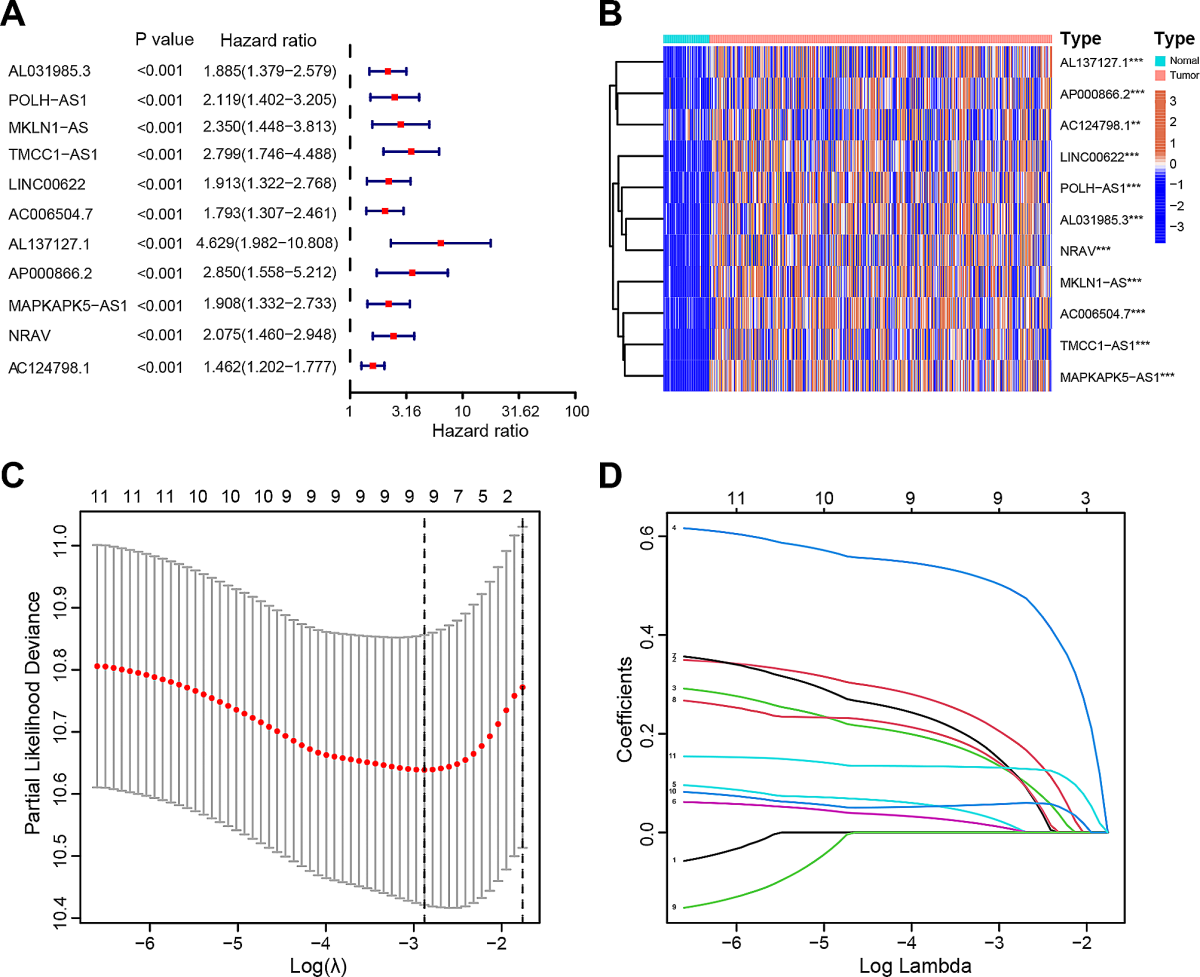
Construction and validation of the prognostic signature of DRLs. (**A**) Forest plot of univariate analysis results showing 11 OS-related DRLs. (**B**) Heatmap showing the expression of 11 OS-related DRLs in the normal and tumor samples. (**C**) Cross-validation plot for the penalty term. (**D**) Diagram for LASSO expression coefficients. ^**^p < 0.01, ^***^p < 0.001

Upon univariate analysis, we identified 11 DRLs from 750 differentially expressed DRLs that exhibited correlations with OS. The forest plot (Fig. 2A), heatmap (Fig. 2B), and Sankey diagram (Fig. S1B) illustrated that all 11 DRLs were upregulated and considered poor prognostic factors for patients with HCC (*p* < 0.001, hazard ratio, HR > 1). In the subsequent Lasso regression analysis aiming at reducing the risk of overfitting (Fig. 2C and D), 9 DRLs were found to be associated with OS. Further multivariate Cox regression narrowed this count to 3 DRLs (POLH-AS1, TMCC1-AS1, AC124798.1), which were used to construct the OS prognostic signature. The risk score for each HCC patient was calculated using the following formula: Risk score = (0.413458729998944 × POLH − AS1 expression) + (0.818274047598138 × TMCC1 − AS1 expression) + (0.248268992114983 × AC124798.1 expression. The correlation heatmap depicted the relationship between DRGs and the three selected DRLs (Fig. S1C).

Patients were stratified into low- and high-risk groups based on the median value of risk scores. As depicted in Fig. S2A-C, the low-risk group exhibited significantly extended survival times compared to the high-risk group across the training set, testing set, and the entire set (*P* < 0.01). Furthermore, the distribution plot of risk score and survival status revealed a positive correlation: higher risk scores corresponded to a higher number of deaths in HCC patients (Fig. S2D-I). The heatmap highlighted elevated expression levels of three DRLs in the high-risk group relative to the low-risk group (Fig. S2J-L). Overall, these findings indicated that patients in the high-risk group experienced worse prognoses.

### Independent prognostic analysis and establishment of a nomogram

**Fig. 3:**
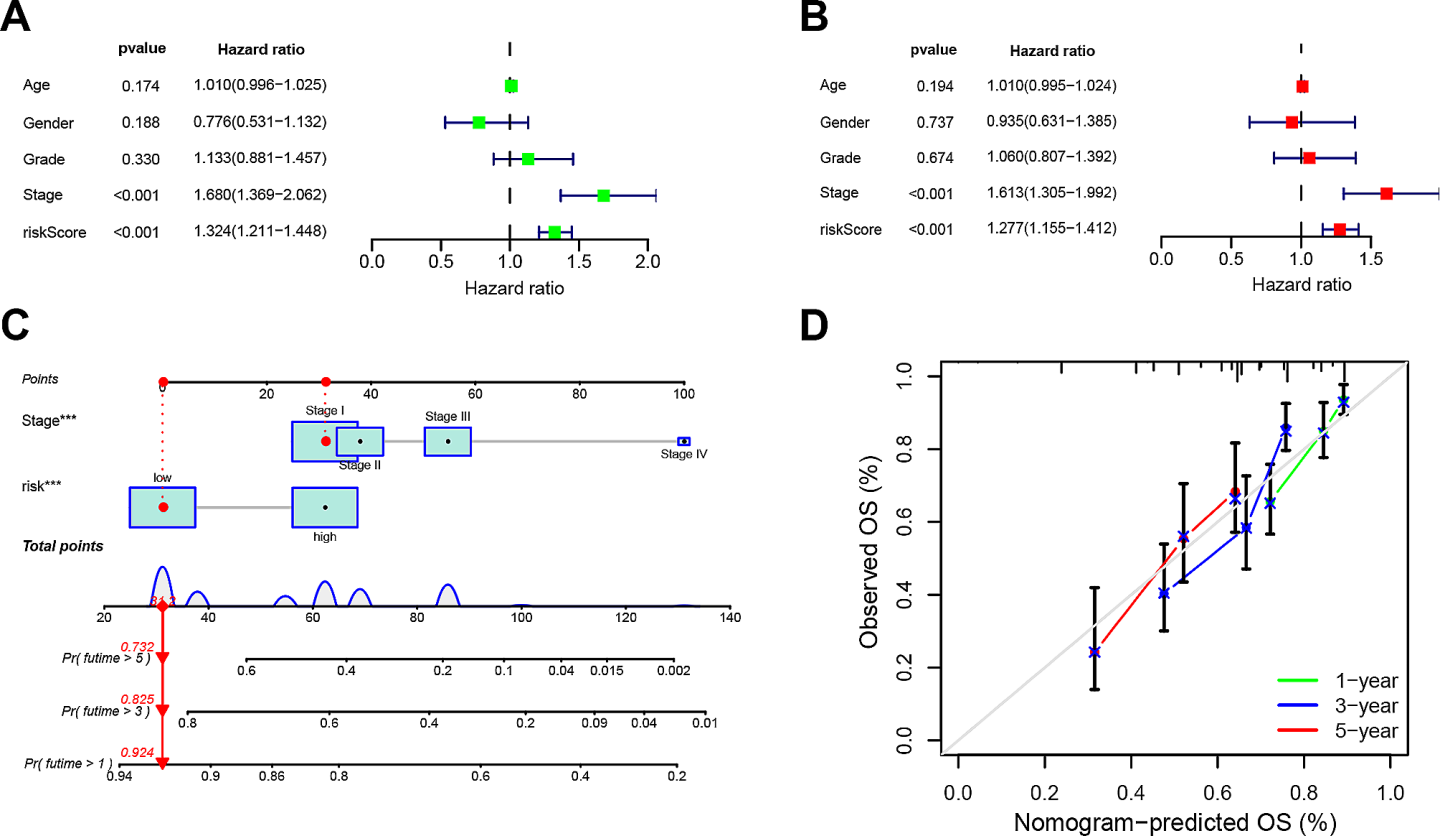
Independent prognostic analysis and establishment of a nomogram. (**A**) Univariate Cox regression analysis of the clinical characteristics and riskScore with the OS. (**B**) Multivariate analysis of the clinical characteristics and riskScore with the OS. (**C**) A nomogram predicting the 1-, 3- and 5-years survival rates of HCC using stage and independent prognostic factors (stage and risk score). (**D**) The calibration curves showing the concordance between the prediction by nomogram and actual survival

To assess the independent prognostic utility of the DRLs signature, we conducted both univariate and multivariate Cox regression analyses. As shown in Fig. 3A, univariate Cox regression analysis demonstrated that the prognostic signature of the three DRLs could predict OS outcomes in HCC patients (HR = 1.324; 95% CI, 1.211–1.448; *p* = 0.001). Multivariate Cox regression analysis further affirmed that the prognostic signature of the three DRLs remained an independent prognostic factor for HCC (HR = 1.277, 95% CI, 1.155–1.412, *p* < 0.001) after adjusting for gender, age, grade, and stage (Fig. 3B).

Subsequently, a nomogram was established employing these independent prognostic factors (stage and risk score) to predict 1-, 3-, and 5-year survival rates for HCC patients (Fig. 3C). Calibration curves were developed to validate the nomogram’s effectiveness in predicting survival rates at 1, 3, and 5 years, demonstrating optimal agreement between nomogram predictions and actual survival outcomes (Fig. 3D).

### Correlation analysis between DRLs signature and clinical characteristics

**Fig. 4:**
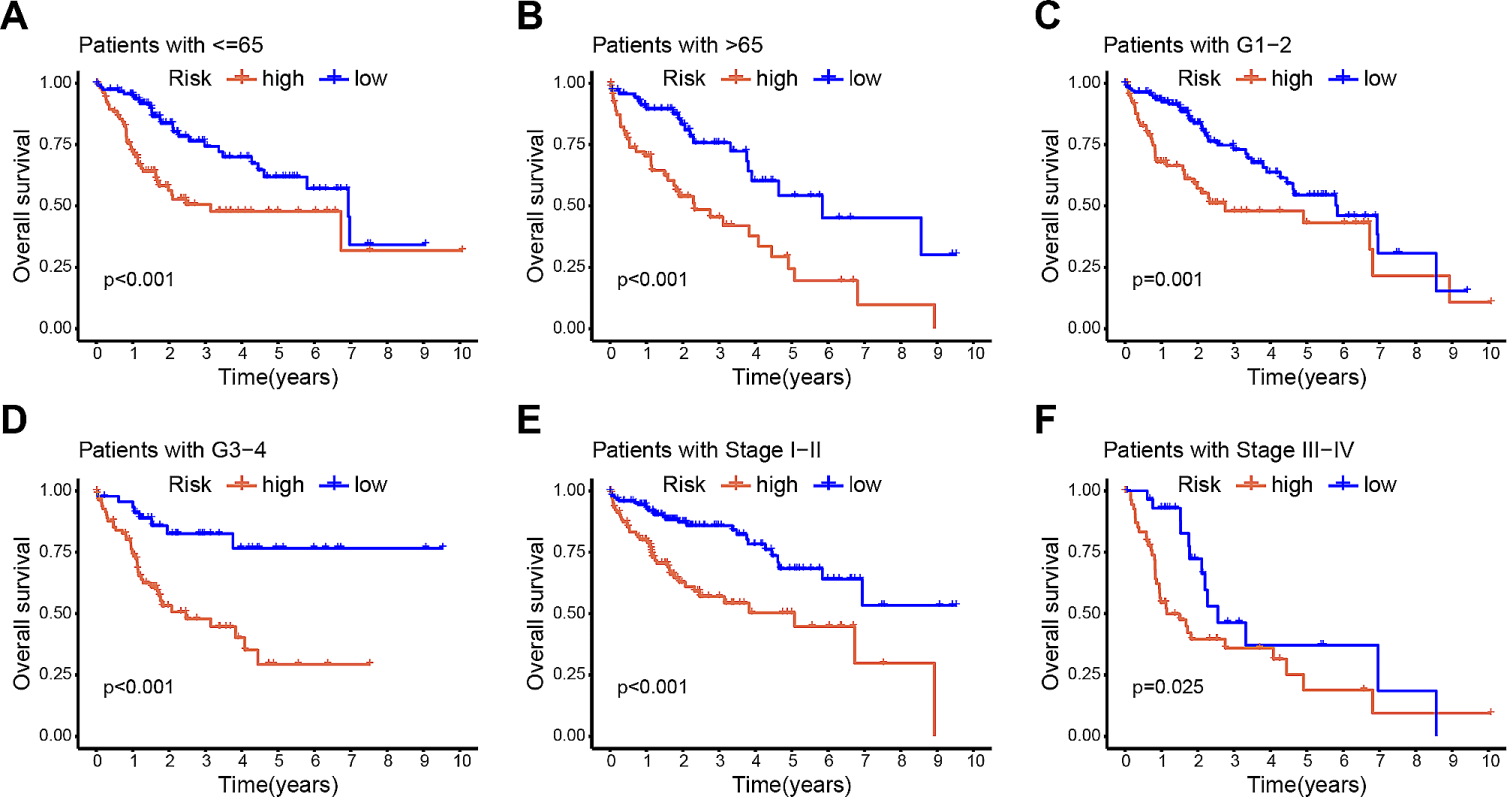
Relationship between the prognostic signature of DRLs and clinical characteristics. (**A**)–(**F**) Kaplan–Meier curve for overall survival in different clinical features such as age (**A**, **B**), grade (**C**, **D**), and stage (**E**, **F**)

To investigate the correlation between the prognostic signature of DRLs and the clinical characteristics of patients with HCC, we examined the relationship between the survival probability and the risk score in different subgroups based on age, grade, and stage. As shown in Fig. 4, the results revealed that patients in the low-risk group had a much higher OS rate than patients in the high-risk group. Furthermore, the concordance index (C-index) of the risk score surpassed that of clinical characteristics, including age, gender, grade, and stage (Fig. S3A).

Additionally, the high-risk group showed a significantly shorter progression-free survival compared to the low-risk group (Fig. S3B). Moreover, the AUC value for the risk grade was 0.754, markedly outperforming the predictive accuracy of individual clinical characteristics, such as age (0.531), gender (0.509), grade (0.499), and stage (0.671) (Fig. S3C). The AUC of the novel DRL signature for 1-, 3-, and 5-year survival rates were 0.754, 0.699, and 0.671, respectively (Fig. S3D). Overall, these findings affirm the reliability of the prognostic signature based on the three DRLs for patients with HCC.

### PCA and functional enrichment analysis

To discern differences between the low- and high-risk groups, we conducted PCA using four expression profiles (entire gene expression profiles, DRGs, DRLs, and the three DRLs risk signature). The results illustrated that the three DRLs exhibited robust discriminatory ability, effectively distinguishing between the low- and high-risk groups (Fig. S4A-D).

**Fig. 5:**
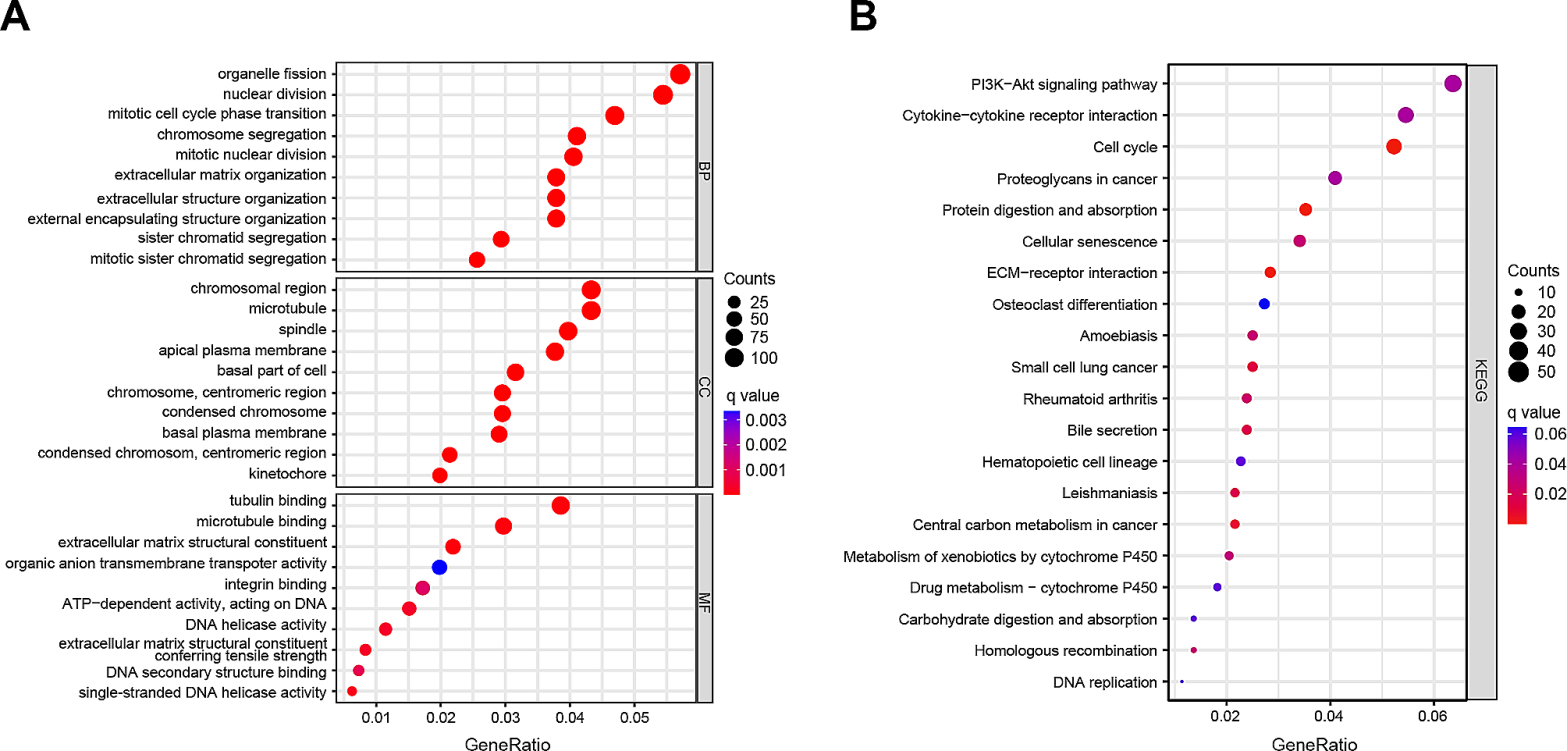
Functional enrichment analyses. (**A**) GO functional enrichment analysis with bubble plot (BP, biological process; CC, cellular component; MF, molecular function). (**B**) KEGG pathway enrichment analysis with bubble plot

Then, we identified 2397 DEGs between the low- and high-risk groups in the TCGA set, comprising 2300 upregulated genes and 97 downregulated genes (|log2 fold change (FC)| > 1 and *p* < 0.05) (Fig. S4E). Functional enrichment analysis was performed to unravel the biological functions of these DEGs. GO analysis revealed significant enrichment in processes such as organelle fission, chromosomal region, and tubulin binding (Fig. 5A). KEGG analysis unveiled enrichment in pathways associated with carcinogenesis, including the PI3K-Akt signaling pathway, cytokine − cytokine receptor interaction, and the cell cycle (Fig. 5B). These results strongly suggest the involvement of DRLs in the development and progression of HCC.

### Evaluation of the immune microenvironment using the DRLs signature

**Fig. 6:**
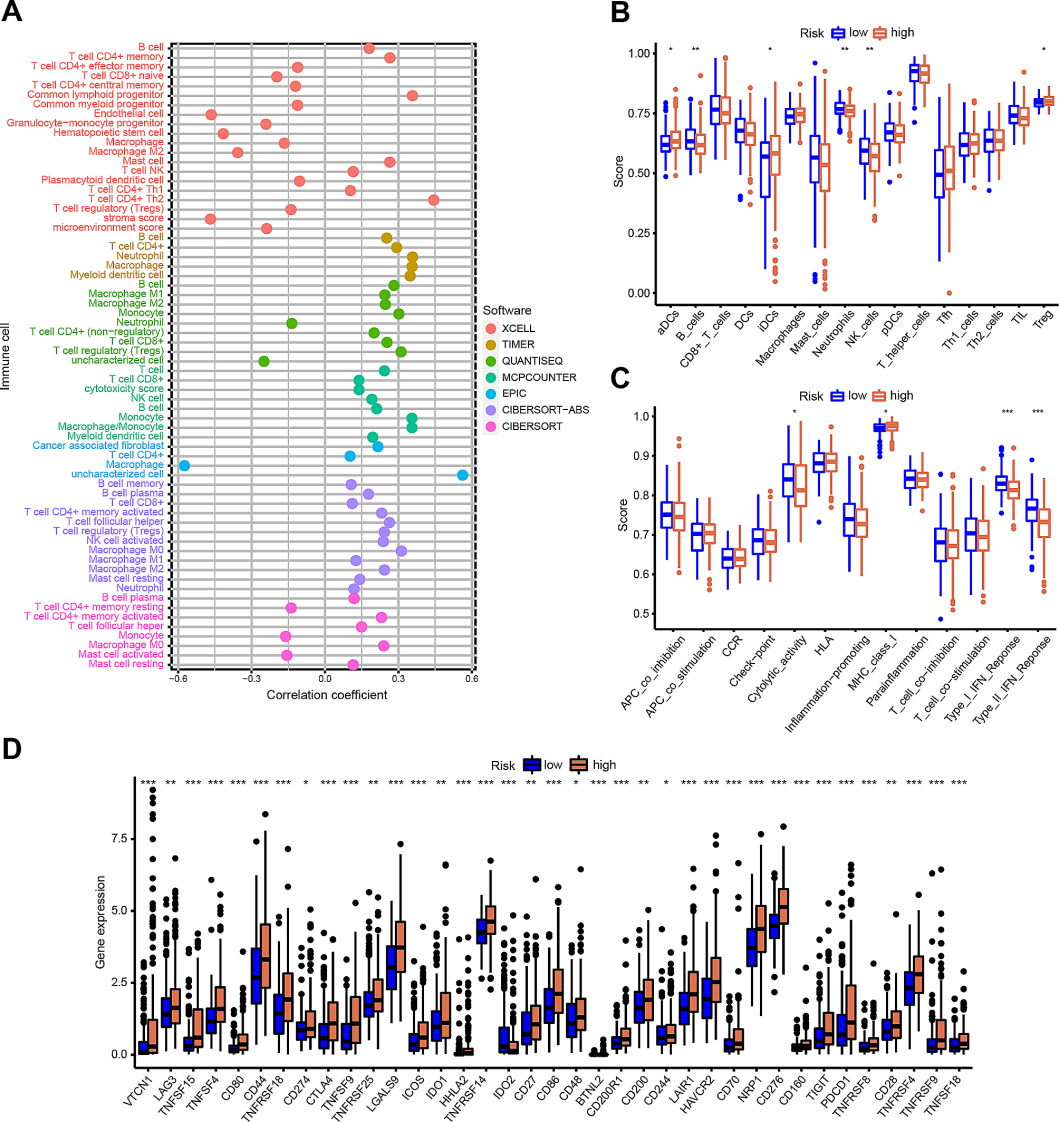
Infiltrations and functions of immune cells between high- and low-risk groups. (**A**) Heatmap for immune infiltration based on TIMER, CIBERSORT, quanTIseq, MCP-counter, xCELL and EPIC algorithms among high- and low-risk groups. (**B**) Single sample gene set enrichment analysis (ssGSEA) showing different extent of immune cell infiltrations in the high- and low-risk groups. (**C**) ssGSEA analyses showing different functions of immune cell in the high- and low-risk groups. (**D**) The expression of immune checkpoint genes between high- and low-risk groups. ^*^p < 0.05, ^**^p < 0.01, ^***^p < 0.001

Immune infiltration stands as a pivotal determinant in countering HCC progression, wielding significant influence over the survival rates of afflicted patients [3, 15]. The heatmap depicting immune responses unveiled substantial correlations between DRLs-scores and various immune cells, encompassing B cells, T cells CD4+, macrophages, and NK cells (Fig. 6A). Employing the ssGSEA method, we delved into the association between DRLs-scores and immune cell subpopulations, unraveling distinct patterns of immune cell infiltrations in the high-risk group characterized by elevated abundance of activated dendritic cells (aDCs), immature dendritic cells (iDCs), and regulatory T cells (Tregs), juxtaposed with diminished levels of B cells, neutrophils, and NK cells (Fig. 6B). Functional disparities in immune cell subpopulations, including cytolytic activity, major histocompatibility complex (MHC) class I, type I interferon (IFN) response, and type II IFN response, were pronounced between the high- and low-risk groups (Fig. 6C). Moreover, immune checkpoint analysis unveiled heightened activation of numerous checkpoints in the high-risk group (Fig. 6D). Collectively, these findings underscored the predictive capability of the DRLs signature regarding the immune microenvironment in HCC patients, holding potential utility in steering individualized immunotherapeutic strategies.

### TMB, TIDE and drug susceptibility analysis

**Fig. 7:**
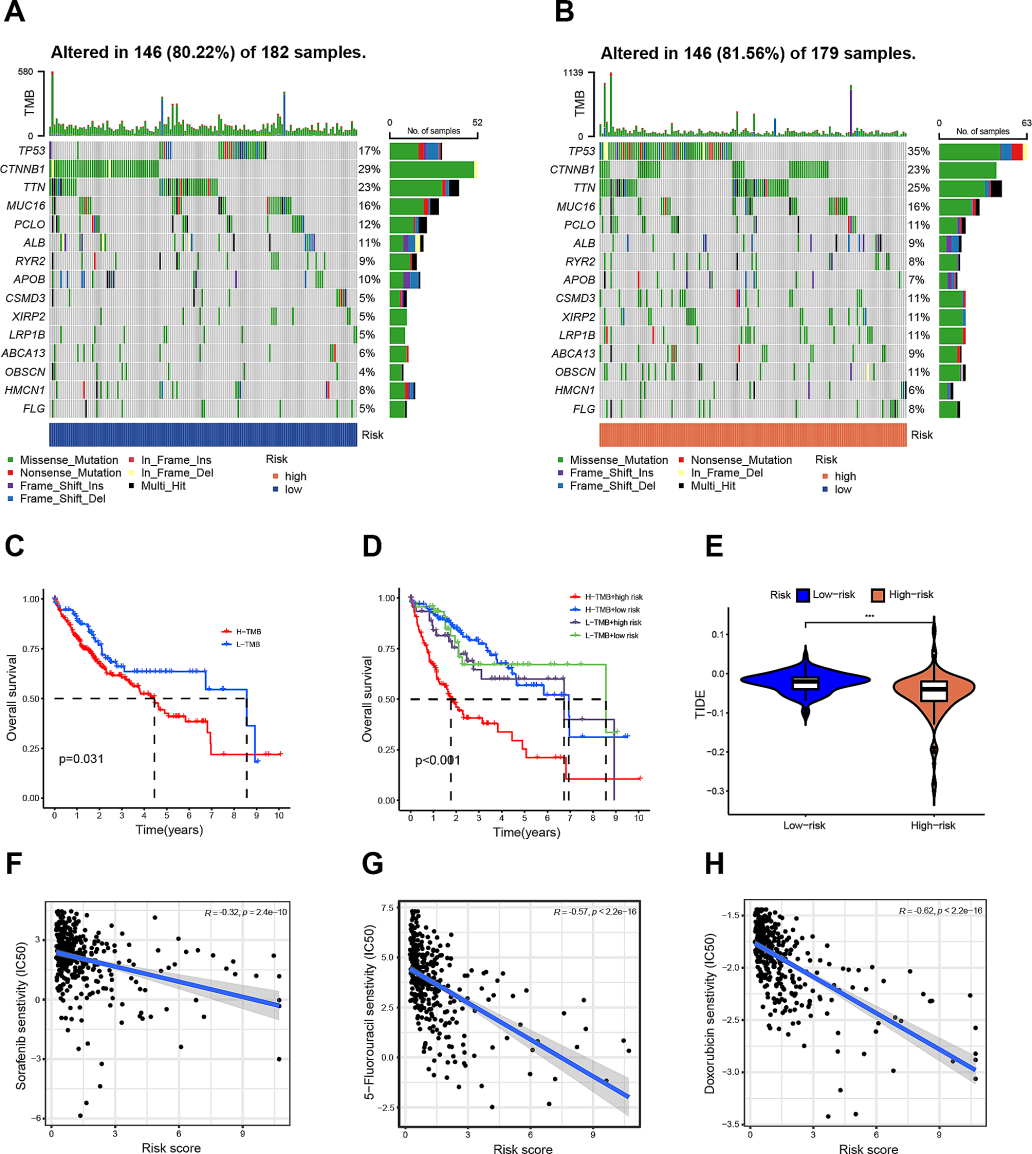
TMB analyses and drug sensitivity between high- and low-risk groups. (**A**-**B**) Waterfall plot displaying the mutation information of the genes with high mutation frequencies in the high- (**A**) and low- (**B**) risk groups. (**C**) Kaplan–Meier curve for OS of patients with HCC in high and low TMB (*p* = 0.031). (**D**) Kaplan–Meier curve for OS of patients with HCC according to the TMB and the risk signature of DRLs. (**E**) The TIDE scores of high- and low-risk groups. (**F**-**H**) The correlation between the risk score of DRLs signature and sensitivity of drugs such as sorafenib (**F**), 5-Fluorouracil (**G**), and doxorubicin (**H**). ^***^p < 0.001

Accumulating evidence suggests a linkage between TMB status and the clinical responsiveness to immunotherapy in HCC [13, 20]. Notably, our findings demonstrated a heightened frequency of mutations in the high-risk group compared to the low-risk group, particularly among the top 15 genes exhibiting the highest mutation rates (Fig. 7A-B). Subsequent categorization of patients into high and low TMB groups based on TMB scores unveiled a superior survival rate in the low TMB group (Fig. 7C). An assessment of the synergistic impact of TMB and DRLs-score groups in prognostic stratification revealed that the high-TMB and high-risk subgroup exhibited the poorest prognosis, while the low-TMB and low-risk subgroup displayed a more favorable prognosis. Importantly, even in instances of high or low TMB, the high-risk subgroup consistently manifested a worse prognosis compared to the low-risk counterpart (Fig. 7D).

Moreover, TIDE analysis was conducted to scrutinize the sensitivity to immunotherapy among HCC patients. Intriguingly, the low-risk group exhibited a higher TIDE score, indicative of a more favorable response to immunotherapy (Fig. 7E). Subsequently, drug susceptibility analysis aimed to discern potential therapeutic agents for HCC treatment based on the IC50 of each drug. The outcomes underscored that patient in the low-score group demonstrated lower IC50 values for anti-cancer drugs such as sorafenib, 5-Fluorouracil, and doxorubicin (Fig. 7F-H). This implies that individuals in the low-risk group might harbor a heightened sensitivity to these three drugs. Collectively, these results advocate for the utility of the DRLs signature as a promising predictor for treatment efficacy in the context of HCC.

### Identifying TMCC1-AS1 as a diagnostic and prognostic biomarker for HCC

In our pursuit of a prognostic biomarker pertinent to DRLs for HCC patients, we initially scrutinized the expression levels of three DRLs (POLH-AS1, TMCC1-AS1, and AC124798.1) in HCC tissues sourced from the TCGA dataset. The findings illuminated a pronounced upregulation of these three DRLs in HCC tissues relative to normal tissues (Fig. S5A-C). Furthermore, diminished expression levels of POLH-AS1, TMCC1-AS1, and AC124798.1 exhibited a significant association with extended overall survival (Fig. S5D-F). Then, our exploration delved into the assessment of the Area Under the Curve (AUC) values for the three DRLs, revealing that TMCC1-AS1 displayed commendable discriminatory prowess for diagnosing patients with HCC (Fig. S5G-I). This underscores the potential of TMCC1-AS1 as a valuable prognostic and diagnostic biomarker for individuals afflicted with HCC.

### Knockdown of TMCC1-AS1 prevented cell proliferation, migration, and invasion in HCC

**Fig. 8:**
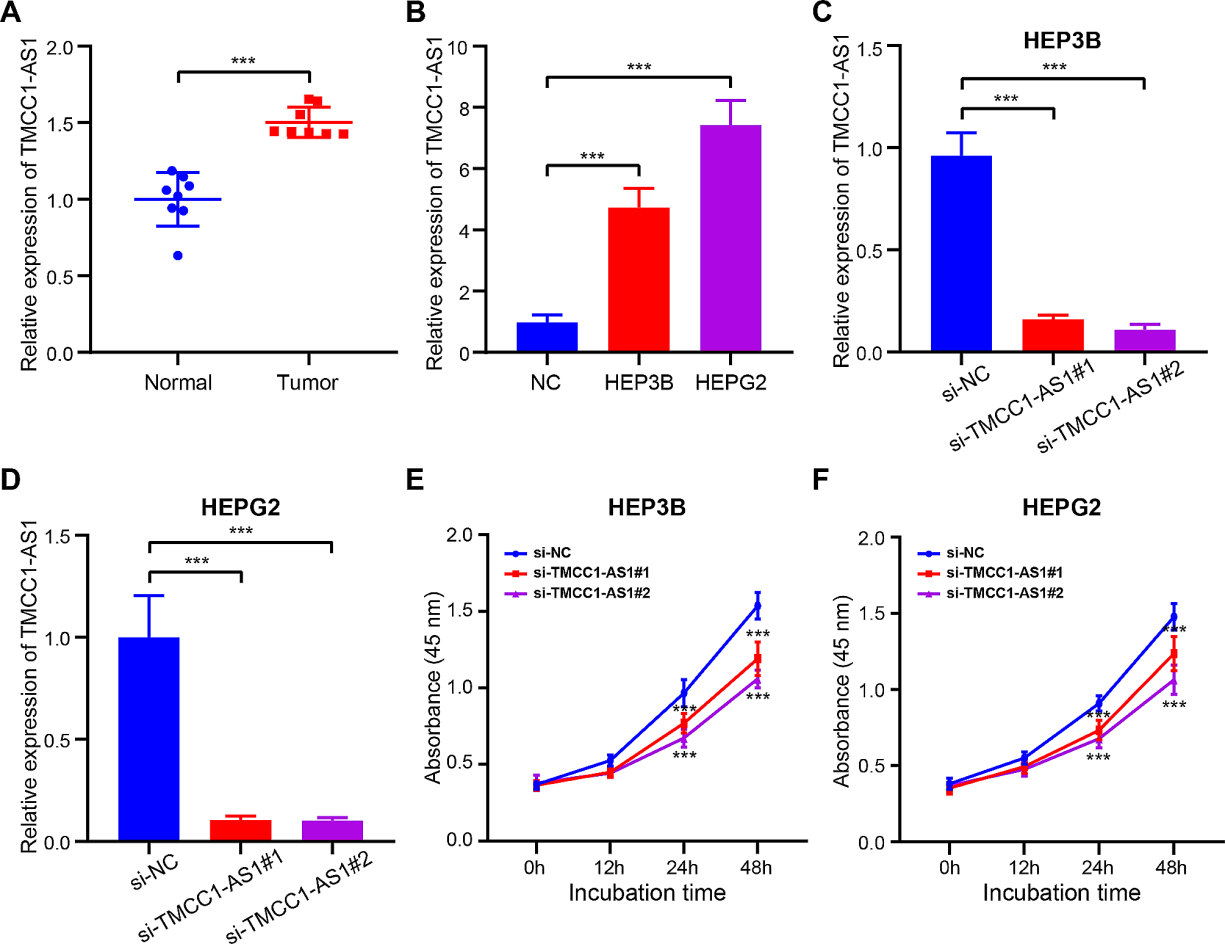
Knockdown of TMCC1-AS1 inhibited cell proliferation in HCC. (**A**) The expression of TMCC1-AS1 was assessed in 8 HCC tissues and 8 normal liver tissues by RT-qPCR assay. (**B**) RT-qPCR analysis showing the expression of TMCC1-AS1 in two HCC cell lines (HEP3B and HEPG2) and a normal liver cell (NC). (**C**-**D**) The efficiency of si-TMCC1-AS1 transfection in HEP3B (**C**) and HEPG2 (**D**) cells was assessed by RT-qPCR. (**E**-**F**) Cell proliferation of HEP3B (**E**) and HEPG2 (**F**) cells transfected with control (si-NC) or si-TMCC1-AS1 was measured via CCK8 assay. Data are presented as the mean ± SDs. ^***^p < 0.001

To further substantiate the functional role of TMCC1-AS1 in HCC, we initially examined its expression levels in both HCC tissues and cell lines (Fig. 8A-B). Notably, TMCC1-AS1 exhibited heightened expression in both HCC tissues and cell lines, namely HEP3B and HEPG2. Subsequent to confirming the elevated expression, we sought to elucidate the impact of TMCC1-AS1 on HCC cell proliferation. Employing siRNA-mediated knockdown of TMCC1-AS1 in HEP3B and HEPG2 cells, we achieved effective silencing, as evidenced by RT-qPCR results (Fig. 8C-D). The growth curves further underscored that the depletion of TMCC1-AS1 significantly impeded the growth of HCC cells, implicating its role in promoting cell proliferation (Fig. 8E-F). Simultaneously, we also investigated the biological functions of POLH-AS1 and AC124798.1, and the ultimate results were consistent with the functions of TMCC1-AS1 described earlier (Fig. S6-7).

**Fig. 9:**
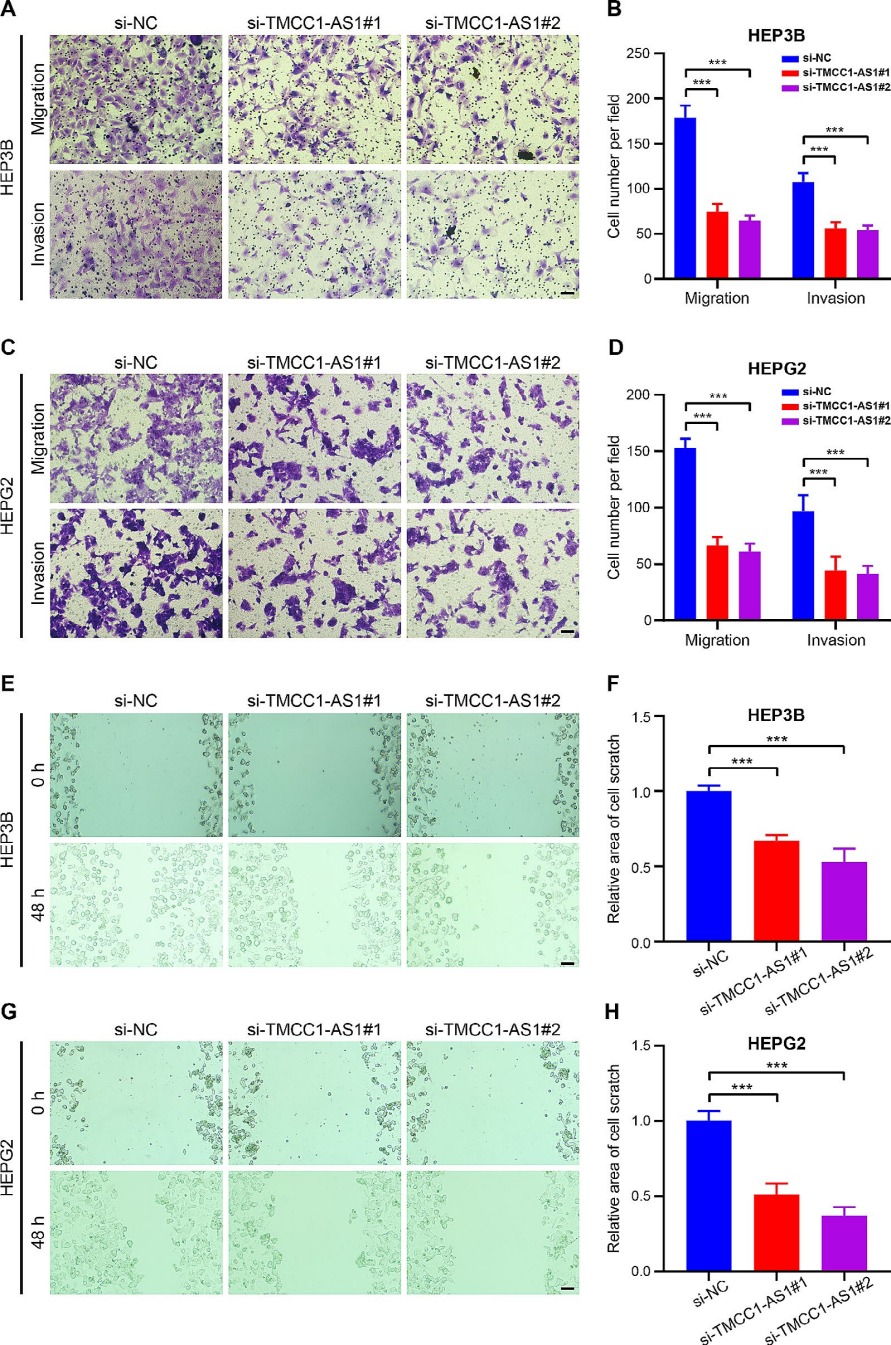
Inhibition of TMCC1-AS1 prevented cell migration and invasion in HCC. (**A**-**D**) Representative data from Transwell migration and invasion assays showing the migratory and invasive capacities of TMCC1-AS1-deficient HEP3B (**A**, **B**) and HEPG2 (**C**, **D**) cells. Scales bar, 100 μM. The data are the means ± SDs and are representative of three independent experiments. (**E**-**H**) Representative data from wound healing migration assays showing HEP3B (**E**, **F**) and HEPG2 (**G**, **H**) cell migration of control cells compared to TMCC1-AS1-depleted cells. Scales bar, 100 μM. Data are presented as the mean ± SDs. The data are the means ± SDs and are representative of three independent experiments. ^***^p < 0.001

Moving beyond proliferation, our investigations extended to migration and invasion capabilities. The Transwell assay unveiled that the inhibition of TMCC1-AS1 markedly curtailed cell migration and invasion in both HEP3B and HEPG2 cells (Fig. 9A-D). Furthermore, the wound healing assay demonstrated that the depletion of TMCC1-AS1 hampered the speed of wound closure in both cell lines (Fig. 9E-H). In summation, these findings strongly suggest that TMCC1-AS1 plays a pivotal role in fueling hepatocellular carcinoma cell proliferation, migration, and invasion in vitro, establishing TMCC1-AS1 as a promising target for therapeutic intervention in HCC.

## Discussion

As the predominant form of primary liver cancer, hepatocellular carcinoma (HCC) significantly jeopardizes the well-being and survival of afflicted individuals due to its elevated morbidity and mortality rates [6]. Recent years have witnessed substantial progress in HCC treatment with the advent of targeted agents like sorafenib and immune checkpoint inhibitors (ICIs) [21]. Nevertheless, the inherent heterogeneity of HCC results in variable treatment outcomes, with only a subset of patients deriving benefit from ICIs and other targeted drugs [22]. Therefore, the identification of innovative biomarkers for prognostication and predicting therapeutic responses holds paramount clinical significance for those grappling with HCC.

Disulfidoptosis has recently garnered extensive attention in tumorigenesis and cancer therapies [23]. It has been proposed that disulfidoptosis-related biomarkers serve as robust prognostic indicators and predictors of antitumor efficacy in various cancers [24]. Additionally, several studies have highlighted the pivotal role of long non-coding RNAs (lncRNAs) in the transport and metabolism of disulfide during tumorigenesis and subsequent tumor progression [17, 25, 26]. Nonetheless, the precise involvement of disulfidoptosis-related lncRNAs (DRLs) in HCC remains elusive, necessitating a comprehensive evaluation of their prognostic significance.

In the current study, we identified 11 prognostically significant DRLs from the TCGA dataset, three of which were selected to construct the prognostic DRLs signature. Regardless of training or testing sets, the DRL signature demonstrated robust efficacy in predicting survival outcomes for HCC patients. Subsequently, we examined the relationship between survival probability and risk score across various clinical characteristics. The results revealed a significantly higher overall survival rate in the low-risk group, irrespective of gender, age, grade, or stage, substantiating the validity of the prognostic DRL signature. Furthermore, we delved into tumorigenesis pathways, the immune microenvironment of HCC, and potential drugs for HCC treatment based on the prognostic signature. Lastly, our investigation unveiled that the inhibition of TMCC1-AS1 suppressed the proliferation, migration, and invasion of hepatocellular carcinoma cells. This study provides valuable insights into the molecular mechanisms underpinning HCC progression and offers potential avenues for personalized therapeutic strategies.

Immunotherapy, an advancing and effective anti-tumor treatment, strengthens the therapeutic effect by regulating the tumor immune microenvironment (TIME) [6]. Presently, TIME is acknowledged for its profound intricacy [14, 27]. Numerous studies have certified that TIME is involved in the process of tumor metastasis, immune escape, and immunotherapy resistance by altering the immune response [27, 28]. In our study, DEGs between different risk groups were enriched in some immune-related biological processes and pathways. Our results unveiled that many immune cells (including B cells, neutrophils, and NK cells) and many functions of immune cell subpopulations (such as cytolytic activity, MHC class I, type I IFN response, and type II IFN response) were significantly different between high- and low-risk groups. Additionally, immune checkpoint-related genes exhibited higher expression levels in the high-risk group compared to the low-risk group. This provides a foundation for discerning responsive patients for immunotherapy. In brief, these results indicated that DRLs signature could reflect the TIME of HCC, which may contribute to personalized immunotherapy and targeted therapy for patients with HCC.

TMB is currently recognized as a valuable biomarker across various cancers, believed to be linked with the efficacy of immunotherapy for HCC [27, 29, 30]. We observed that the proportion of gene mutations differed significantly between the two groups and that the high-risk group had higher frequency of mutations than the low-risk group in the top 15 genes with the highest mutation rates. Specifically, it was found that patients in the high-risk group had a significantly higher frequency of TP53 mutation (35% vs. 17%). TP53 is a typical tumor suppressor, and its mutation leads to the development and progression of many types of tumors, including HCC [29, 31]. This is consistent with our results where the low TMB group had a higher survival rate than the high TMB group.

Recent studies have elucidated that epigenetics, transport processes, regulated cell death, and the tumor microenvironment are involved in the development of drug resistance in HCC [32, 33]. To enhance the treatment of patients with HCC, we evaluated the drug sensitivity of different anticancer drugs in the treatment of patients with HCC in different DRL-score groups. Based on IC50 values, the drugs of sorafenib, 5-Fluorouracil, and doxorubicin showed better responses in the low-score group than in the high-score group. These findings indicated that DRLs signature could be used as a potential predictor for the efficacy of medical treatment of HCC. Moreover, the occurrence of drug resistance may be reduced by regulating the DRLs; this brings new breakthroughs for the choice of individual therapeutic strategies.

The study outcomes revealed 11 DRLs influencing the survival of HCC patients, with POLH-AS1, TMCC1-AS1, and AC124798.1 selected to compose the prognostic signature. Among them, the expression of POLH-AS1 was confirmed to be upregulated in HCC tissues based on RT-qPCR [27]. Fang et al. investigated a novel risk model with POLH-AS1 for predicting the prognosis of HCC [6]. In addition, Cui et al. identified TMCC1-AS1 as a valuable resource for novel biomarker and therapeutic target identification in HCC [34]. Furthermore, Zhu et al. constructed a prognostic signature with AC124798.1 to predict the prognosis of pancreatic adenocarcinoma [35]. However, few studies have investigated whether these three DRLs contribute to the progression of HCC.

To substantiate the prognostic potential of the identified DRLs, we conducted further investigations using the TCGA dataset. Our findings indicated elevated expression of these DRLs in HCC tissues, correlating with poorer survival outcomes. Notably, TMCC1-AS1 exhibited a higher AUC compared to POLH-AS1 and AC124798.1, suggesting its potential as a more promising biomarker for HCC diagnosis and prognosis. Subsequently, we elucidated the biological roles of TMCC1-AS1 in HCC, revealing significantly lower expression in NC compared to HEP3B and HEPG2 cells. Inhibition of TMCC1-AS1 effectively impeded HCC cell growth, migration, and invasion. These results align with Zhao et al., who observed TMCC1-AS1 as a prognostic biomarker for HCC patients [36]. In summary, TMCC1-AS1 appears to play a role in promoting HCC cell growth and migration in vitro, suggesting its potential as a therapeutic target.

Nevertheless, the study has inevitable limitations. Firstly, the sample data solely originated from TCGA databases, lacking clinical information from external cohorts. Secondly, the absence of comprehensive clinical follow-up data hinders thorough validation and assessment of the prognostic model’s clinical value. Finally, the precise mechanisms through which TMCC1-AS1 influences HCC growth, invasion, and migration remain incompletely understood, necessitating further comprehensive experimental investigations.

## Conclusions

Conclusively, the DRLs signature demonstrated promising prognostic value, offering insights into the immune microenvironment and potential therapeutic avenues for HCC. Particularly, TMCC1-AS1 showed potential as a novel prognostic biomarker and therapeutic target for HCC.

### Electronic supplementary material

Below is the link to the electronic supplementary material.

**Supplementary Material 1:** Supplementary Figure S1

**Supplementary Material 2:** Supplementary Figure S2

**Supplementary Material 3:** Supplementary Figure S3

**Supplementary Material 4:** Supplementary Figure S4

**Supplementary Material 5:** Supplementary Figure S5

**Supplementary Material 6:** Supplementary Figure S6

**Supplementary Material 7:** Supplementary Figure S7

**Supplementary Material 8:** Supplementary Figure legends and Supplementary Table S1-S2

## Data Availability

Data from this study can be found in the TCGA databases (http://cancergenome.nih.gov).

## Abbreviations

AUC: Area under the receiver operating characteristic
DEGs: Differentially expressed genes
DRGs: Disulfidptosis-related genes
DRLs: Disulfidptosis-related lncRNAs
GO: Gene Ontology
GSEA: Gene set enrichment analysis
KEGG: Kyoto Encyclopedia of Genes and Genomes
LASSO: Least absolute selection operator
OS: Overall survival
PCA: Principal component analysis
ROC: Receiver operating characteristic
TCGA: The Cancer Genome Atlas
TIME: Tumor immune microenvironment
TMB: Tumor mutation burden
