# Supplementary figures and images for: Integrating bioinformatics and experimental validation to unveil disulfidptosis-related lncRNAs as prognostic biomarker and therapeutic target in hepatocellular carcinoma
Source: Cancer Cell Int. 2024 Jan 13;24:30. doi: 10.1186/s12935-023-03208-x (PMC10788009; doi:10.1186/s12935-023-03208-x)

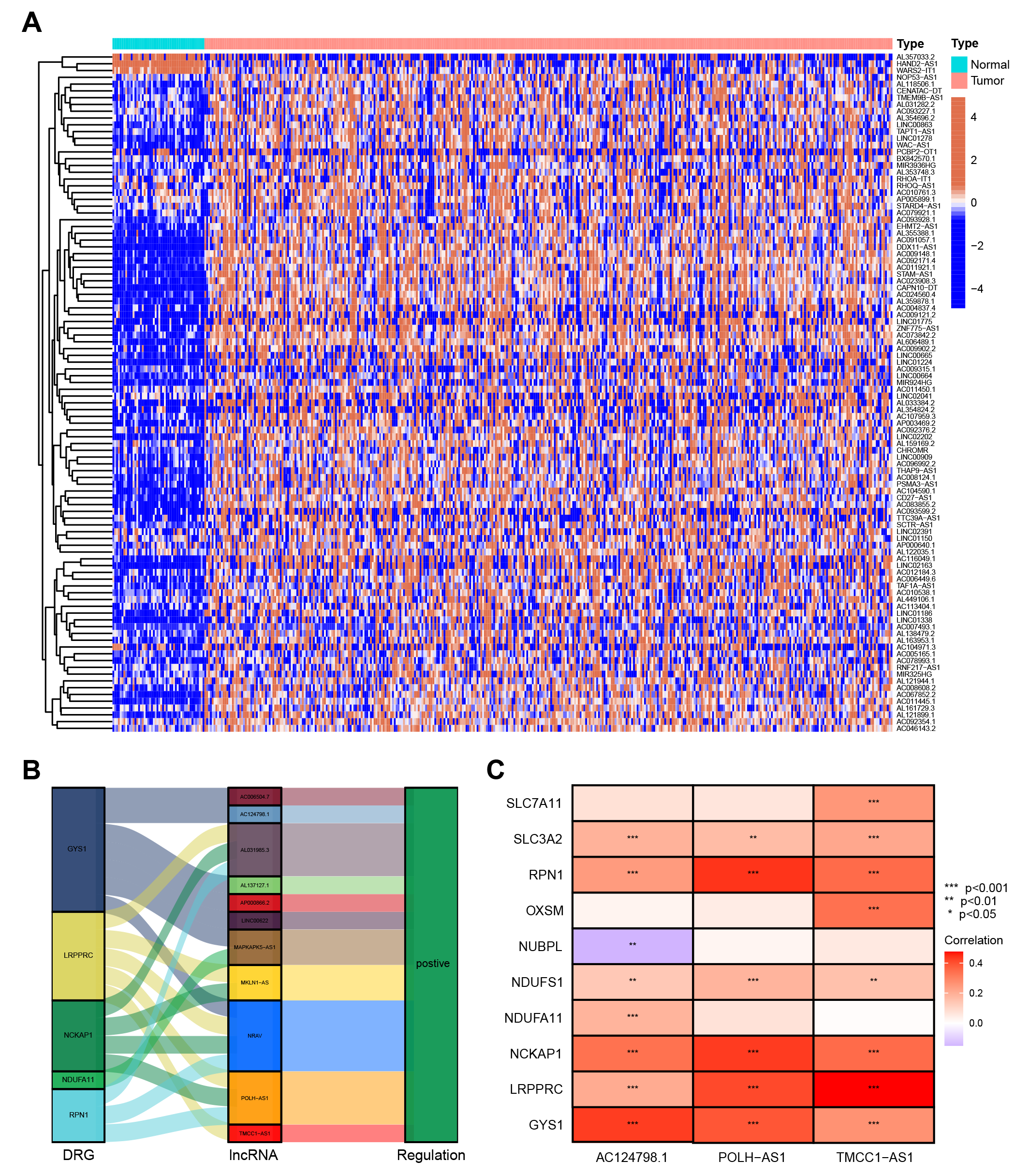

Supplement: Supplementary file 1 — Supplementary Material 1: Supplementary Figure S1 [file 12935_2023_3208_MOESM1_ESM.png]

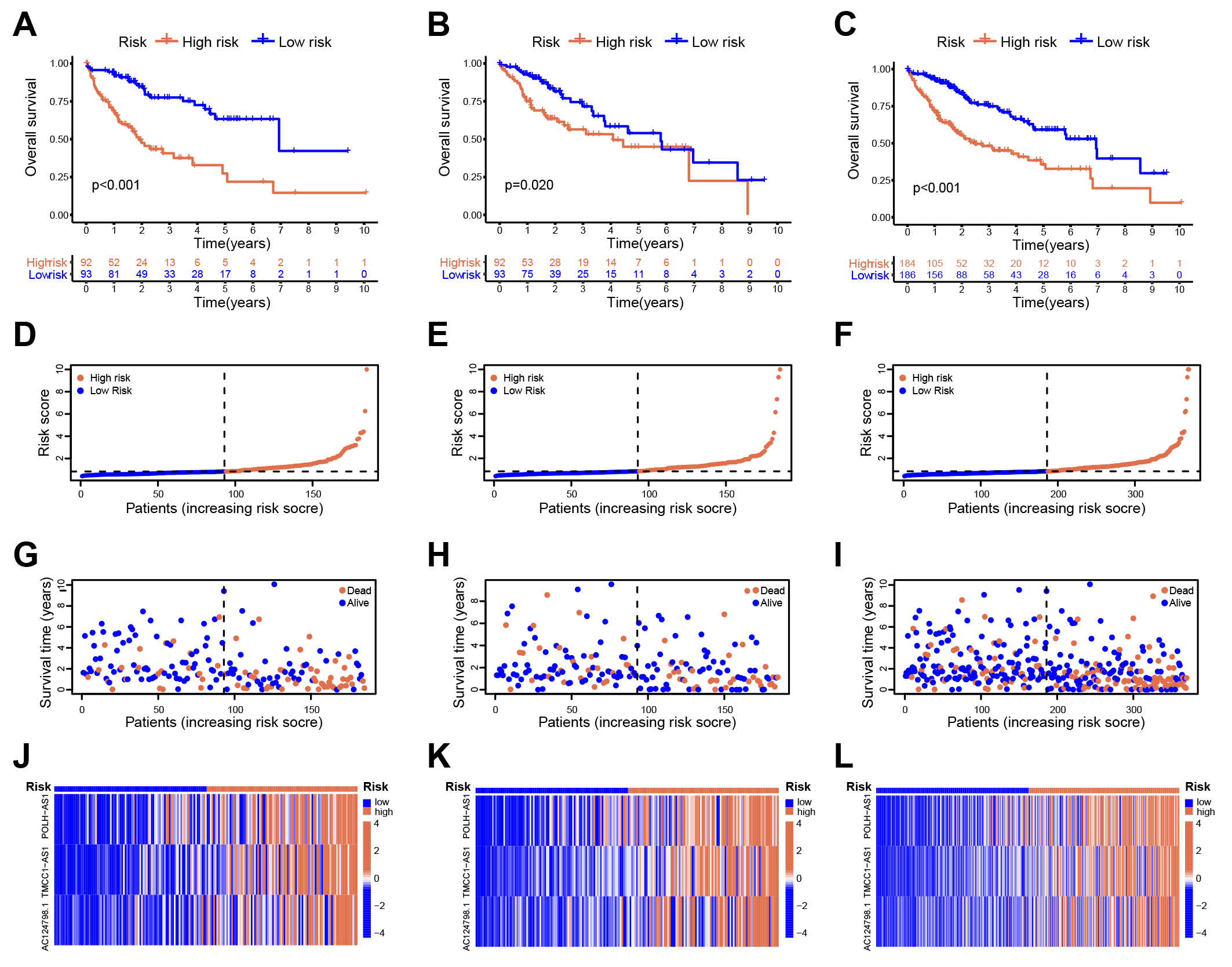

Supplement: Supplementary file 2 — Supplementary Material 2: Supplementary Figure S2 [file 12935_2023_3208_MOESM2_ESM.png]

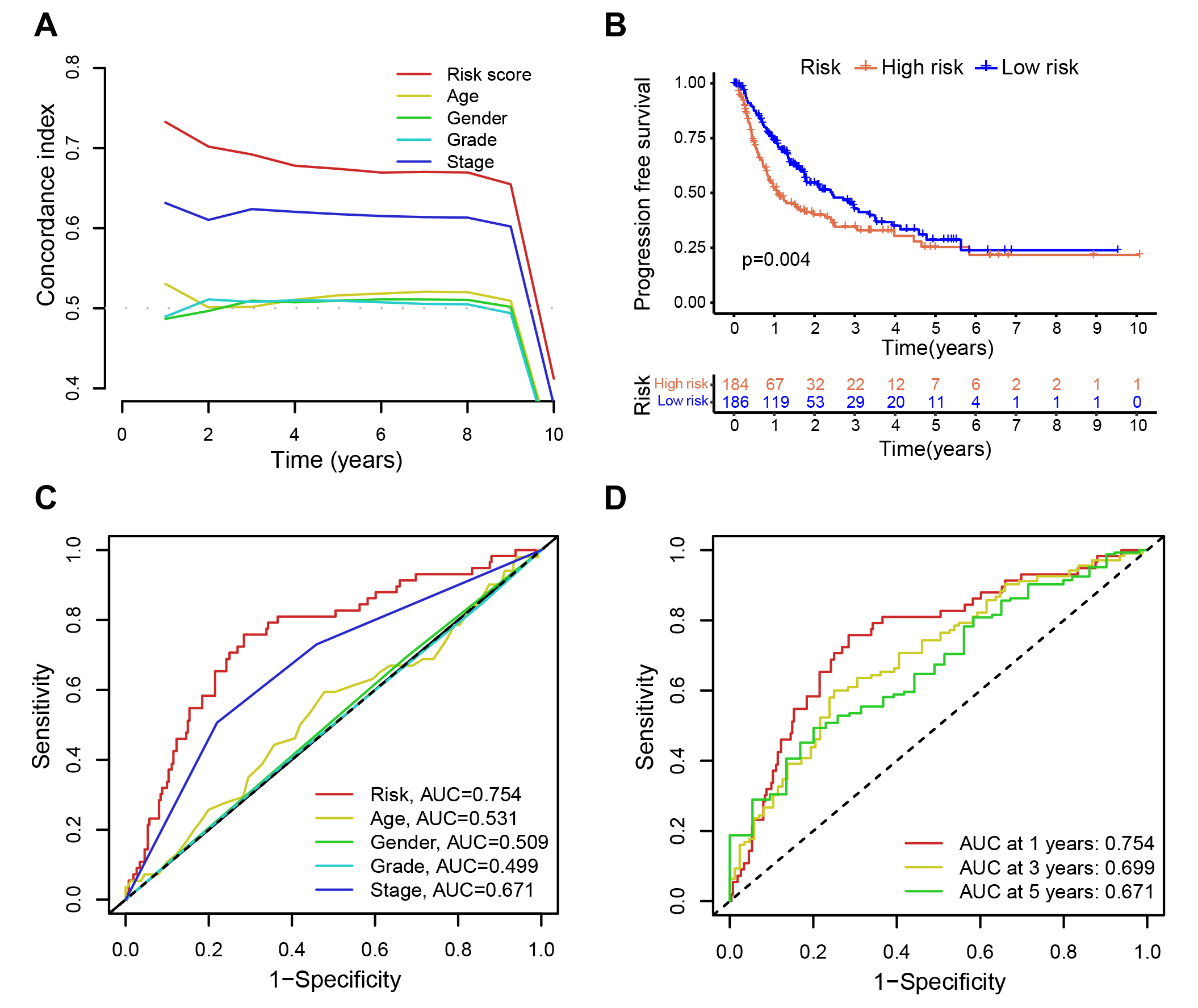

Supplement: Supplementary file 3 — Supplementary Material 3: Supplementary Figure S3 [file 12935_2023_3208_MOESM3_ESM.png]

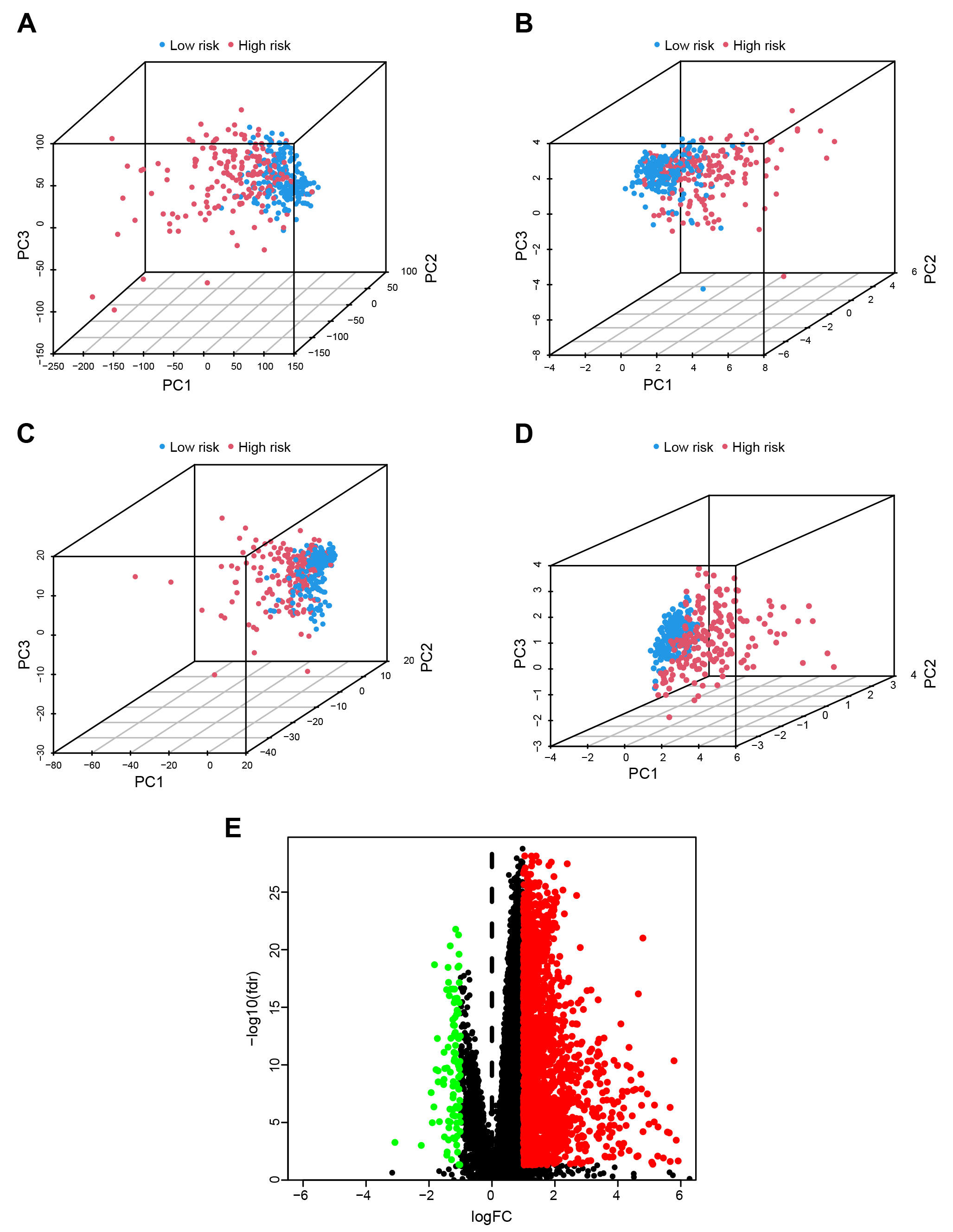

Supplement: Supplementary file 4 — Supplementary Material 4: Supplementary Figure S4 [file 12935_2023_3208_MOESM4_ESM.png]

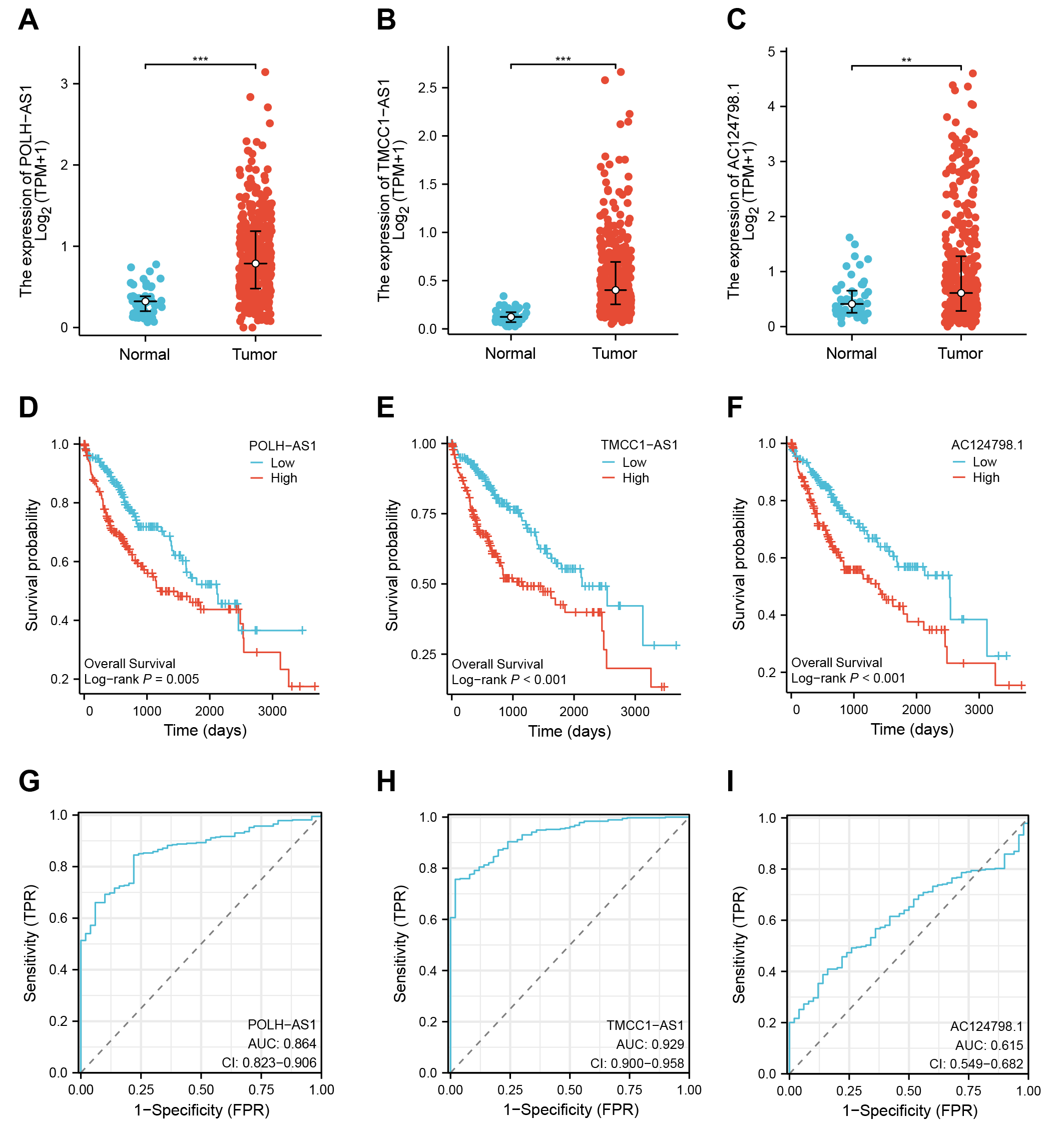

Supplement: Supplementary file 5 — Supplementary Material 5: Supplementary Figure S5 [file 12935_2023_3208_MOESM5_ESM.png]

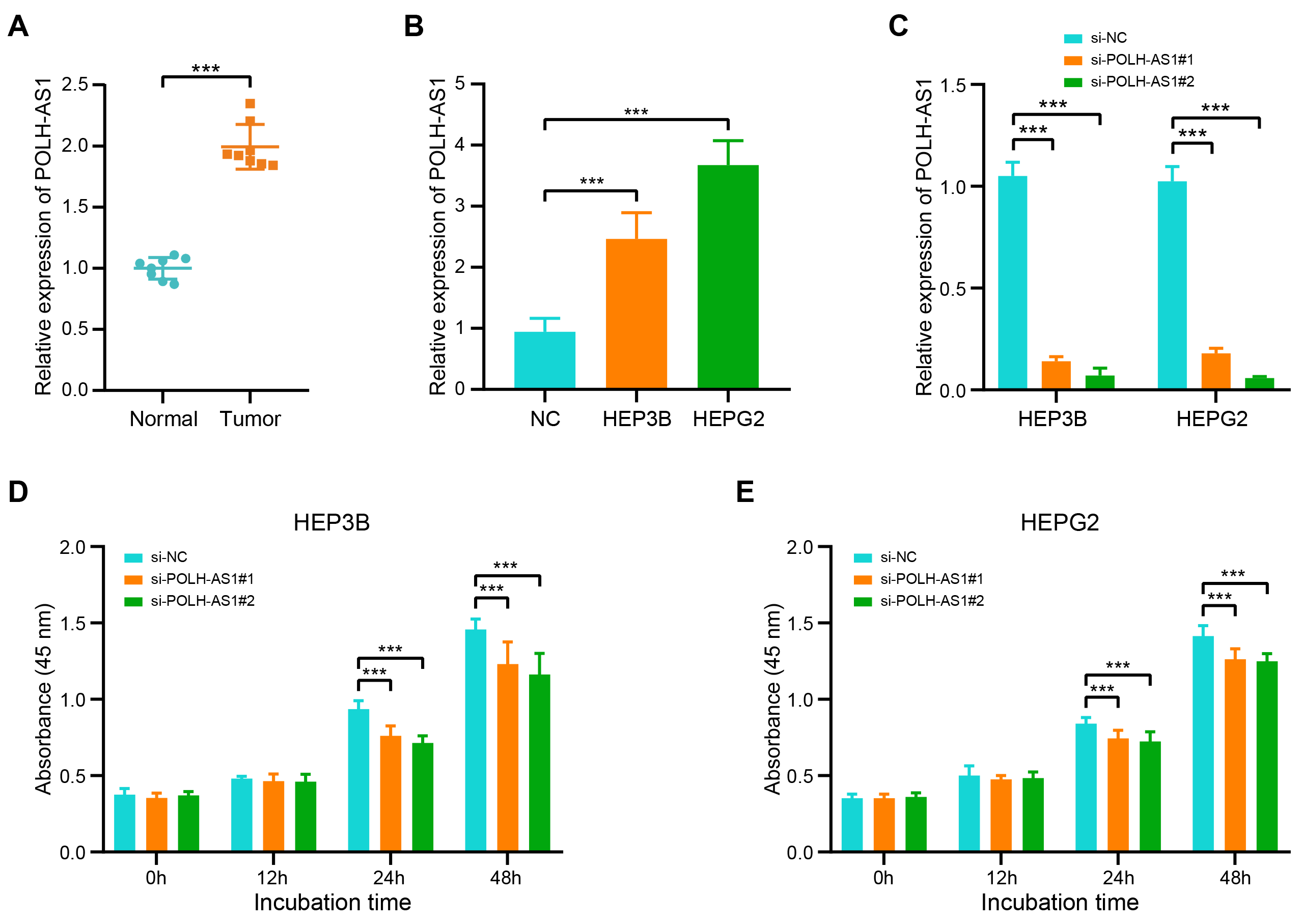

Supplement: Supplementary file 6 — Supplementary Material 6: Supplementary Figure S6 [file 12935_2023_3208_MOESM6_ESM.png]

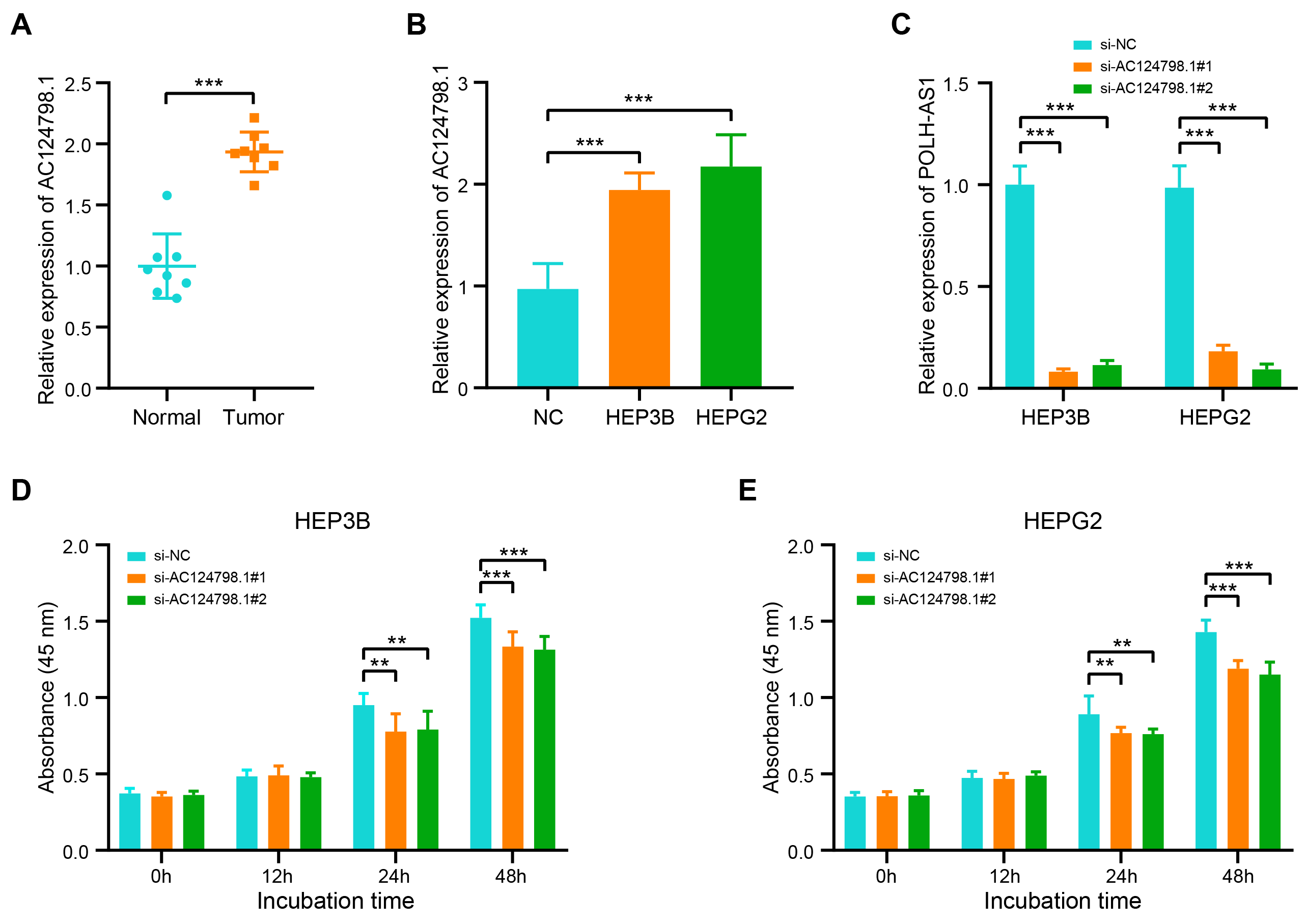

Supplement: Supplementary file 7 — Supplementary Material 7: Supplementary Figure S7 [file 12935_2023_3208_MOESM7_ESM.png]
