# Supplementary material for: Integrating bioinformatics and experimental validation to unveil disulfidptosis-related lncRNAs as prognostic biomarker and therapeutic target in hepatocellular carcinoma
Source: Cancer Cell Int. 2024 Jan 13;24:30. doi: 10.1186/s12935-023-03208-x (PMC10788009; doi:10.1186/s12935-023-03208-x)
Supplement: Supplementary file 8 — Supplementary Material 8: Supplementary Figure legends and Supplementary Table S1-S2 [file 12935_2023_3208_MOESM8_ESM.docx]

**Supplementary figure legends**

**Fig. S1 Identification of disulfidptosis-related lncRNA (DRLs) in patients with hepatocellular carcinoma (HCC)**

(A) Heatmap showing the differential expression of DRLs between normal and tumor samples (|log2 fold change (FC)| > 1 and p < 0.05). (B) Sankey diagram showing the interaction between disulfidptosis-related genes and 11 OS-DRLs. (C) Heatmap showing the relationship between DRGs and three DRLs.

**Fig. S2 The validation of the prognostic signature of DRLs**

(A)–(C) Kaplan–Meier curves for OS in the training sets (A), testing sets (B), and entire set (C). (D)–(F) Distribution of DRLs model-based riskScore for the training sets (D), testing sets (E), and entire set (F). (G)–(I) Patterns of survival time and survival status ranked by riskScore in the training sets (G), testing sets (H), and entire set (I). (J)–(L) The heatmap showing the display levels of the three lncRNA for each patient in the training sets (J), testing sets (K), and entire set (L).

**Fig. S3 ROC curves and AUC values**

(A) The concordance index of the risk factors including risk scores, age, gender, grade, and stage. (B) Kaplan–Meier curves for progression free survival in the high- and low-risk groups. (C) The AUC values of the risk factors including risk scores, age, gender, grade, and stage. (D) The AUC of the DRL signature for 1-, 3-, 5-year survival rates of HCC.

**Fig. S4 Principal component analysis (PCA)**

(A)-(D) PCA showing the distribution differences between the high- and low-risk groups based on the entire gene expression (A), disulfidptosis genes (B), disulfidptosis-related lncRNAs (C), and the risk signature of three DRLs (D). (E) Heatmap showing 2397 DEGs between the low- and high-risk groups of the TCGA set, containing 2300 upregulated genes and 97 downregulated genes (|log2 fold change (FC)| > 1 and p < 0.05).

**Fig. S5 Validation of the three DRLs expressions and prognoses in HCC**

(A)-(C) The expression levels of POLH-AS1, TMCC1-AS1 and AC124798.1 in HCC from TCGA dataset. (D)-(F) K-M survival curve of overall survival (OS) of POLH-AS1, TMCC1-AS1 and AC124798.1 in HCC from TCGA dataset. (G)-(I) The ROC curve of POLH-AS1, TMCC1-AS1 and AC124798.1 in HCC from TCGA dataset.

**Fig. S6 Knockdown of POLH-AS1 inhibited cell proliferation in HCC.**

(A) The expression of POLH-AS1 was assessed in 8 HCC tissues and 8 normal liver tissues by RT-qPCR assay. (B) RT-qPCR analysis showing the expression of POLH-AS1 in two HCC cell lines (HEP3B and HEPG2) and a normal liver cell (NC). (C) The efficiency of si-POLH-AS1 transfection in HEP3B and HEPG2 cells was assessed by RT-qPCR. (D-E) Cell proliferation of HEP3B (D) and HEPG2 (E) cells transfected with control (si-NC) or si-POLH-AS1 was measured via CCK8 assay. Data are presented as the mean ± SDs. ***p < 0.001.

**Fig. S7 Inhibition of AC124798.1 prevented cell proliferation in HCC.**

(A) RT-qPCR analysis showing the expression of AC124798.1 in 8 HCC tissues and 8 normal liver tissues. (B) RT-qPCR analysis showing the expression of AC124798.1 in two HCC cell lines (HEP3B and HEPG2) and a normal liver cell (NC). (C) RT-qPCR analysis showing the efficiency of si-AC124798.1 transfection in HEP3B and HEPG2 cells. (D-E) CCK8 assay showing the cell proliferation of HEP3B (D) and HEPG2 (E) cells transfected with control (si-NC) or si-AC124798.1. Data are presented as the mean ± SDs. ***p < 0.001.

**Supplementary Table S1: Primer list of PCR.**

| Gene Name | Forward primer | Reverse primer |
| --- | --- | --- |
| GAPDH | CGACTTATACATGGCCTTA | TTCCGATCACTGTTGGAAT |
| TMCC1‑AS1 | AGCGAGGGATCGAGTTGAGA | TAGTCATGTCCCCGTTGGTG |
| POLH-AS1 | CTGCAGCCTCTAGCTTCACAT | CTGCCCAGGGAAGCTTGTGA |
| AC124798.1 | TTTTGTTGGAGTGGGGGCTT | ATCCTGGCCCAAAATCCCTG |

**Supplementary Table S2: Sequence of the applied plasmid.**

| Gene Name | Sequence |
| --- | --- |
| si-NC | CGAACUCACUGGUCUGACC |
| si-TMCC1‑AS1#1 | TTGAAACTTAAGCCCATC |
| si-TMCC1‑AS1#2 | TAAGCCGGTTATTGTACAT |
| si-POLH-AS1#1 | AACAUAUAUCAAAUCUAUCUC |
| si-POLH-AS1#2 | AUUGUUCAGCAGCAAAAGCAA |
| si-AC124798.1#1 | CGTCTACTCTCTATCTCCATGAATT |
| si-AC124798.1#2 | GATGGACACTTAGCTTGCTTCCAAA |
